# Microrna let-7: an emerging next-generation cancer therapeutic

**DOI:** 10.3747/co.v17i1.356

**Published:** 2010-02

**Authors:** D. Barh, R. Malhotra, B. Ravi, P. Sindhurani

**Affiliations:** * Centre for Genomics and Applied Gene Technology, Institute of Integrative Omics and Applied Biotechnology, Nonakuri, Purba Medinipur, India; † Maharani Lakshmi Ammanni College for Women, Bangalore University, Malleshwaram, Bangalore, India; ‡ Functional Genomics Unit, Institute of Genomics and Integrative Biology, Council of Scientific and Industrial Research, Delhi, India

**Keywords:** Let-7, microrna, cancer therapy, let-7 regulation, future medicine

## Abstract

In recent years, various rna-based technologies have been under evaluation as potential next-generation cancer therapeutics. Micrornas (mirnas), known to regulate the cell cycle and development, are deregulated in various cancers. Thus, they might serve as good targets or candidates in an exploration of anticancer therapeutics. One attractive candidate for this purpose is let-7 (“lethal-7”).

Let-7 is underexpressed in various cancers, and restoration of its normal expression is found to inhibit cancer growth by targeting various oncogenes and inhibiting key regulators of several mitogenic pathways. *In vivo,* let-7 administration was found effective against mouse-model lung and breast cancers, and our computational prediction supports the possible effectiveness of let-7 in estrogen receptor (er)–positive metastatic breast cancer. Data also suggest that let-7 regulates apoptosis and cancer stem cell (csc) differentiation and can therefore be tested as a potential therapeutic in cancer treatment. However, the exact role of let-7 in cancer is not yet fully understood. There is a need to understand the causative molecular basis of let-7 alterations in cancer and to develop proper delivery systems before proceeding to therapeutic applications. This article attempts to highlight certain critical aspects of let-7’s therapeutic potential in cancer.

## 1. INTRODUCTION

Micrornas (mirnas) are natural non-coding rnas of approximately 22 nucleotides (nt) in size. They regulate genes post-transcriptionally by binding to a site in the 3′ untranslated region (utr) of target messenger rnas (mrnas). Identification of an mirna target involves base pairing with the target site, which is mostly imperfect in the case of animals. However, a perfect pairing in a 7-nt region at the 5′ end of mirna, called the seed region, is essential for target identification 1.

The mirnas are known to regulate cellular processes such as stem-cell differentiation, heart development 2–4, insulin secretion 5, apoptosis 6,7, aging 8,9, and immunity 10,11, among other processes. It is therefore not surprising that mirnas are differentially expressed in several pathophysiologic conditions including, for instance, Alzheimer disease 12,13, Parkinson disease 14, cardiovascular diseases 4,15,16, the Cowden and Down syndromes 17,18, and various cancers 19.

Let-7 was first discovered and well studied in *Caenorhabditis elegans,* in which it regulates developmental timing 20–23 (larval stage 4–to–adult transition 20,24) and stage-specific neuromuscular tissue development 25. Let-7 has orthologs in various species. In *Drosophila,* let-7 plays a role in determining the timing for cell-cycle exit, metamorphosis, neuromuscular Junction development, juvenile-to-adult-stage transition, and adult behaviour 26,27. The zebrafish ortholog of let-7 is prominently expressed in nervous tissue, indicating its certain role in neural development 28. In the adult newt, let-7 regulates transdifferentiation and regeneration of lens and inner ear-hair cells 29.

Little is known about the function of let-7 in mammalian development and normal physiology. In the mouse, let-7 is involved in neural lineage specificity of embryonic stem cells, brain development 30, and mammary epithelial progenitor cell maintenance by induction of loss of self-renewal 31. In humans, 12 genomic loci encode the let-7 family members (let-7a-1, -2, -3; let-7b; let-7c; let-7d; let-7e; let-7f-1, -2; let-7g; let-7i; mir98). Human let-7 is upregulated during embryonic cell differentiation 32, but the roles it plays in normal physiology are mostly unknown.

Human let-7 family members are found to be downregulated in several cancers, with a few exceptions (Table I); restoration of normal expression prevents tumorigenesis 37,44,45,52. Let-7 therefore acts as a tumour suppressor and a regulator of terminal differentiation and apoptosis. This finding implies that let-7 can possibly be used as a next-generation cancer therapeutic. But, to date, the mechanism of let-7 deregulation, and its precise role in tumorigenesis, is not fully understood, creating a hurdle to effectively using this mirna in cancer therapy.

This article presents an overview of let-7 and discusses the critical issues that must be explored to develop a let-7–based therapeutic strategy against various cancers.

## 2. DISCUSSION

### 2.1 Biogenesis and Mechanism of Action

**FIGURE 1:**
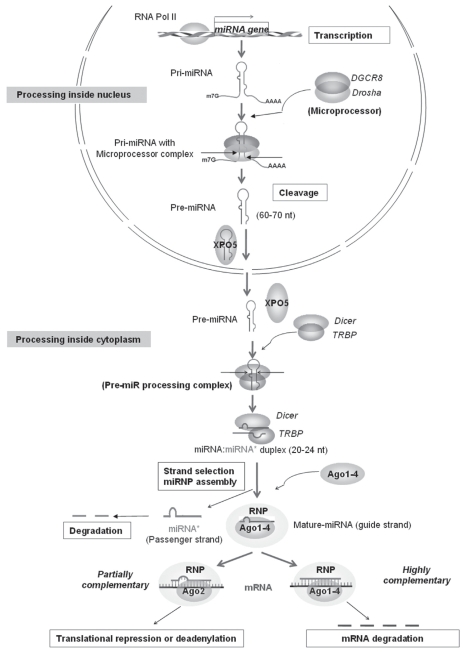
The most-accepted model of microrna (mirna) biogenesis and its mechanism of action. For detail, see text. rna Pol ii = rna polymerase ii; Pri-mirna = primary transcripts of mirna; DGCR8 = DiGeorge syndrome critical region gene 8; Drosha = class 2 rnase iii enzyme; XPO5 = exportin 5; Dicer = formal symbol DICER1 (dicer 1, ribonuclease type iii); TRBP = now labelled TARBP2P [tar (hiv-1) rna binding protein 2 pseudogene]; Ago1–4 = Argonaute-1 to -4 [symbol EIF2C1, 2, 3, 4 (eukaryotic translation initiation factor 2C, 1–4)]; rnp = ribonucleoprotein; mrna = messenger rna.

The biogenesis of let-7 is similar to that of other mirnas. The first step in mirna biogenesis is transcription from the mirna transcription unit by rna polymerase ii to produce a primary transcript called pri-mirna. The pri-mirna is processed by the microprocessor complex containing an rnase iii–like enzyme, Drosha, and its cofactor, a double-stranded rna binding protein, Dgcr8, to produce an approximately 60–70 nt pre-mirna (precursor mirna). The pre-mirna is then transported to cytoplasm by exportin 5 (*XPO5*), in a Rangtp (*ras-*related nuclear protein–guanosine triphosphate complex)–dependent way, where it is cleaved by Dicer (a cytoplasmic rnase iii), to generate an imperfect mirna:mirna* duplex of approximately 21–24 nt. One of the strands (the “guide strand”) from the duplex is then incorporated into Argonaute (Ago)–containing ribonucleoprotein (rnp) complex; the other strand (the “passenger strand”) is degraded. However, there are cases in which both strands of the duplex are detected in the cell 53. The mirna–Ago rnp complex causes posttranscriptional regulation of genes, in which mirna is used as a tether to guide the complex to the specific mrna. The exact mechanism by which the mirnp complex regulates expression of the target remains unclear. Various models try to explain this mechanism 1. Figure 1 shows a general model.

### 2.2 Regulation of Let-7

**FIGURE 2:**
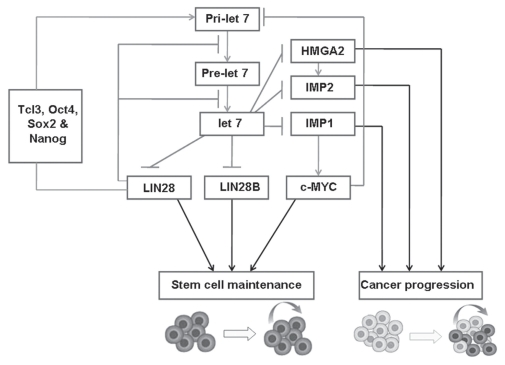
Regulatory circuits of microrna (mirna) let-7. The loop consists of pluripotency promoting factors {LIN28 [lin-28 homolog (*Caenorhabditis elegans*)], OCT4 [now labelled POU5F1 (pou class 5 homeobox 1)], SOX2 [sry (sex determining region Y)–box 2], NANOG [Nanog homeobox], and TCL3 [now labelled TLX1 (T-cell leukemia homeobox 1)]}, oncofetal genes [HMGA2 (high mobility group at–hook 2) and imps (insulin-like growth factor 2 mrna-binding proteins)], and oncogene MYC. For detail, see text. Pri-let 7 = primary transcripts of let-7; LIN28B = lin-28 homolog B (*C. elegans*).

Expression of let-7 is regulated at various stages of its biogenesis and also depending on cell type. Similarly, let-7 regulates many transcription factors that play important roles in regulation of the cell cycle, cell differentiation, and apoptosis. Many of the factors controlling the expression of let-7 form regulatory circuits with the factors being regulated by such expression. These regulatory circuits—such as double-negative feedback loops and so on—are salient network motifs in development and differentiation. *LIN28, POU5F1, SOX2, NANOG, TLX1, HMGA2, MYC,* and *IMP*s are known to form such regulatory loops (Figure 2).

#### 2.2.1 Regulation of Let-7 by Pluripotency-Promoting Factors in Embryonic and Cancer Stem Cells

*LIN28,* which maintains the undifferentiated state of embryonic cells, is a well-known target of let-7 and is downregulated by let-7 during developmental commitment 54,55. Lin28 was recently shown to act as a posttranscriptional repressor of let-7 biogenesis, binding to the loop portion of the pri–let-7 hairpin and the stem part of pre–let-7 and thereby inhibiting its processing. Lin28 and Lin28B also inhibit processing of let-7 by mediating terminal uridylation of let-7 precursors 56. What is unclear is whether the regulation by Lin28 occurs at the Drosha or Dicer processing step 55,57–59. Lin28 induces pri–let-7 expression through induction of other pluripotency-promoting factors such as Pou5F1, Sox2, Nanog, and Tlx1 60, thus regulating let-7 expression at multiple levels.

The early embryonic oncofetal gene *HMGA2* is involved in the self-renewal and maintenance of adult stem cells. It is highly expressed in hematopoietic and fetal neuronal stem cells 61,62, and the low levels of let-7 in stem cells inversely correlate with *HMGA2* expression. Thus, the undifferentiated state is maintained 63. In differentiated tissues, *HMGA2* is downregulated because of the high expression of let-7 61, and during induced differentiation, ectopic expression of let-7 reduces *ras* and *HMGA2* expression, leading to inhibition of cell proliferation and induction of apoptosis. Therefore, *HMGA2* is a direct target of let-7 64.

Like normal stem cells, cancer stem cells (slowly dividing tumour-initiating cells) exhibit low levels of let-7 and possess unlimited self-renewal capability and pluripotency, allowing them to repopulate and metastasize 65,66. It has been proposed that, during carcinogenesis, the let-7–targeted embryonic genes, which are otherwise not expressed in adult tissues, are re-expressed because of loss of let-7 control. This reprogramming promotes de-differentiation and cancer progression 67. A good example is that of *HMGA2,* which is undetectable in most differentiated tissues, but highly expressed in various cancers, including neuroblastoma and pancreatic, lung, and thyroid cancers 68–71. Breast cancer stem cells are also devoid of let-7, but abundantly express *HMGA2* and *ras* 36 (Figure 2).

#### 2.2.2 Regulatory Circuit Between Myc and Let-7

*IMP1* is another oncofetal gene that is expressed only during early fetal life 72,73 and is re-expressed in several cancers 74. It is selectively expressed in young, but not in old, hematopoietic stem cells 75. *IMP1* regulates stem cell functions by stabilizing insulin-like growth factor 2 and C-*myc* mrnas 76,77, and the phenotype of stem cells from the *IMP1* knockout mouse resembles that of cells from the *HMGA2-*deficient mouse 73,78. Let-7 targets *IMP1*, and therefore indirectly acts as a negative regulator of *MYC* expression 64,79,80. It has been shown that Myc binds directly to let-7 promoter and downregulates its transcription 81. Thus, an indirect feedback circuit exists between let-7 and Myc (Figure 2).

### 2.3 Let-7 Targets Multiple Oncogenes and Components of Cell Cycle, Cell Proliferation, and Apoptosis

**FIGURE 3:**
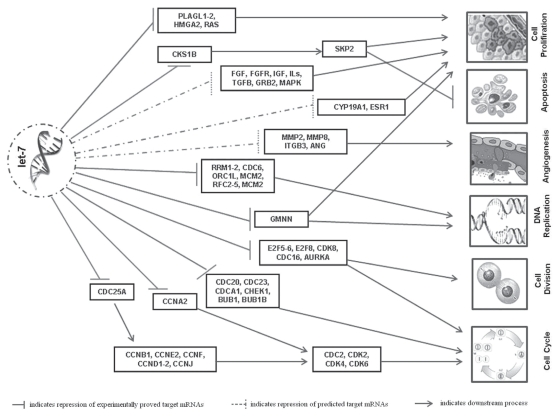
Let-7 targets various key components of mitogenic and tumorigenic pathways to exert its tumour suppressor activity. Pathways include cell cycle, cell division, cell proliferation, dna replication, angiogenesis, and apoptosis. PLAGL1, 2 = pleomorphic adenoma gene-like 1, 2; CKS1B = cdc28 protein kinase regulatory subunit 1B; SKP2 = S-phase kinase-associated protein 2 (p45); fgf, fgfr = fibroblast growth factor and fibroblast growth factor receptor; igf = insulin-like growth factor; il-s = interleukin S; tgfb = transforming growth factor β; GRB2 = growth factor receptor-bound protein 2; mapk = mitogen-activated protein kinase; CYP19A1 = cytochrome P450, family 19, subfamily A, polypeptide 1; ESR1 = estrogen receptor 1; MMP2, 8 = matrix metallopeptidases 2, 8; ITGB3 = integrin β3; ANG = angiogenin; RRM1, 2 = ribonucleotide reductases M1 and M2; CDC6 = cell division cycle 6 homolog (*Saccharomyces cerevisiae*); ORC1L = origin recognition complex, subunit 1-like (yeast); MCM2 = minichromosome maintenance complex component 2; RFC2–5 = replication factor C (activator 1) 2–5; GMNN = geminin, dna replication inhibitor; E2F5, 6, 8 = e2f transcription factors 5, 6, 8; CDK8 = cyclin-dependent kinase 8; CDC16 = cell division cycle 16 homolog (*S. cerevisiae*); AURKA = aurora kinase A; CDC25A = cell division cycle 25 homolog A (*Schizosaccharomyces pombe*); CCNA2 = cyclin A2; CDC20, 23 = cell division cycle 20 and 23 homologs (*S. cerevisiae*); CDCA1 = (now labelled NUF2) NDC80 kinetochore complex component, homolog (*S. cerevisiae*); CHEK1 = chk1 checkpoint homolog (*S. pombe*); BUB1, 1B = budding uninhibited by benzimidazoles 1 and 1 β homologs (yeast); CCNB1, D1, D2, E2, F, J = cyclins B1, D1, D2, E2, F, J; CDC2 = cell division cycle 2, G1 to S and G2 to M; CDK2, 4, 6 = cyclin-dependent kinases 2, 4, 6; mrna = messenger rna.

Apart from targeting oncogenes (*ras, MYC, HMGA2*, and so on) as already discussed, let-7 regulates several key components of the cell cycle and cell proliferation. Microarray analysis of hepatocellular carcinoma (HepG2) and lung cancer (A549) cell lines revealed that let-7 inhibits multiple cell-cycle- and proliferation-associated genes, including cyclin A2 (*CCNA2*), *CDC34,* Aurora A [*AURKA* (formerly *STK6*)] and B [*AURKB* (formerly *SKT12*)] kinases, *E2F5, CDK8,* and *PLAGL2,* among others 46. In HepG2 cells, let-7 directly represses *CCNA2, CDC25A, SKP2, AURKA, CDC16, CCND1,* and *CDK6,* among others. Let-7 also inhibits several dna replication machinery components (*ORC1L; RRM1, 2*; and so on) and transcription factors [*E2F6, CBFB, PLAGL2, SOX9, GZF1* (formerly *ZNF336*), *YAP1, GTF2I, ARID3A,* and so on]. Surprisingly, that study also showed that let-7 represses several tumour suppressor genes (*BRCA1, BRCA2, FANCD2,* and *PLAGL1,* among others) and checkpoint regulators (*CHEK1, BUB1, BUB1B, MAD2L1,* and *CDC23,* among others). Our recent *in silico* analysis shows that let-7 may potentially target er signalling and angiogenic pathways by targeting key molecules of these cascades 82. Various targets of let-7 are listed in Table II and shown in Figure 3.

Apoptosis regulatory functions of let-7 have recently been reported in both human and mouse. Let-7 targets Casp3 in the A431 and HepG2 cell lines, and inhibits doxorubicin- and paclitaxel-induced apoptosis 85. In NIH3T3 mouse fibroblast cells, let-7 is involved in ultraviolet B–induced apoptosis by modulating Casp3, Bcl2, Map3k1, and Cdk5 86.

### 2.4 Emerging Role of Let-7 in Cancer Diagnosis and Therapy

The facts discussed here indicate that let-7 acts as a tumour suppressor by targeting various oncogenes and key components of the cell cycle and developmental pathways. Most reports reveal that let-7 is frequently underexpressed (Table I) and that the chromosomal region of human let-7 is frequently deleted in many cancers 87. Similarly, in more differentiated tumour cells, let-7 is expressed at higher levels, and its target oncogenes (*HMGA2* and *ras*) are downregulated. Thus, loss of let-7 expression is a marker for less differentiated cancer 88, and expression levels are also found to be effective prognostic markers in several cancers 40,46,88. In lung cancer, reduced let-7 expression was also found to significantly correlate with shortened postoperative survival regardless of disease stage 45.

From the therapeutic viewpoint, let-7 is attractive molecule for preventing tumorigenesis and angiogenesis 89; it is a potential therapeutic in several cancers that underexpress let-7. Let-7 replacement was found to inhibit anchorage-independent growth and cell-cycle progression in melanoma cells by repressing regulators of the cell cycle and cell proliferation such as cyclins A, D1, and D3 and *CDK4* 47. Together with *TP53, ras* and *MYC* have been implicated as key oncogenes in lung cancer. The reduced expression of let-7 in lung cancer directly correlates with upregulation of oncogene *ras*; introduction of let-7 represses *ras* and *MYC* translation by targeting the related mrnas 45,46. In both lung and hepatocellular carcinomas, replacement or restoration of normal expression levels of let-7 inhibits cancer growth by repressing multiple cell-cycle and proliferation pathways, together with *ras* and *MYC* 37,44,45,52 (Table II). Intranasal let-7 administration was found effective in reducing tumour growth in a K-*ras* mutant mouse model of lung cancer 90. Similarly, restoration of let-7 restrains the growth and proliferation of colon and hepatic cancers 40,80. Transfection of let-7 in a Burkitt lymphoma cell line downregulates *MYC* and reverts *MYC-*induced cell growth 38. Ectopic expression of let-7 inhibits cell proliferation by directly repressing the *HMGA2* oncogene in lung cancers 52,83 and uterine leiomyoma 84.

Induced expression of let-7 in breast cancer cells targets *HMGA2* and H-*ras* 36, and in a mouse model of breast cancer, exogenous let-7 delivery suppresses cell proliferation, mammosphere formation, and the population of undifferentiated cells by downregulating both of the foregoing oncogenes 35,36. In our *in silico* analysis, we recently showed that, apart from repressing *MYC, ras,* and *HMGA2,* let-7 may also target *CYP19A1, ESR1,* and *ESR2,* thereby potentially blocking estrogen signalling in er-positive breast cancers. Similarly, by repressing angiogenin, fibroblast growth factor, transforming growth factor, interleukin 6, and matrix metallopeptidase 2, let-7 may prevent growth, angiogenesis, and metastasis in breast cancer 82 (Table II).

### 2.5 Limitations of Let-7–Based Therapy

#### 2.5.1 Limitations Because of Limited Knowledge of Let-7 Biology

Although restoration of normal let-7 expression proves beneficial, limited knowledge concerning its transcriptional and processing control during biogenesis and its exact role in tumorigenesis make it difficult to directly apply let-7 as a therapeutic. It is necessary to know whether downregulation of let-7 in tumours is a primary or secondary phenomenon during tumorigenesis. Supporting the csc hypothesis, we agree with the opinion that epigenetic downregulation of let-7 in cscs leads to upregulation of oncofetal genes (*HMGA2* and *LIN28,* among others) and, thereby, to loss of differentiation and tumorigenesis. In that scenario, downregulation of let-7 is the primary event, a view that can be supported by observation of where in ovarian cancer let-7 is hypermethylated 48.

Because mirnas act on the 3′ utr of target mrnas, it is important to determine how efficiently let-7 will work as a therapeutic, because 3′ utr truncated oncogenes may be prevalent in neoplasia. Grimm *et al.* 91 reported that delivery of adeno-associated virus (aav)–mediated recombinant pre-mirnas causes death in mice from severe liver cytotoxicity. Details of the immunogenic and cytotoxic effects of let-7 therefore need to be explored so that such side effects can be minimized in an effective treatment strategy. Similarly, we proposed that let-7 may be involved in an as-yet-unknown regulatory network of mirnas that resembles the gene regulatory network involving transcription factors. Therefore, anti-mirna oligo-based knockdown of let-7 inhibitory mirnas is not currently possible.

#### 2.5.2 Limitations in Delivery Methods and Systems

Lack of an appropriate, safe, and effective delivery method for let-7 is another drawback of possible therapy. Biological vectors such as aav and lentivirus may be used for targeted delivery 92, but standardization of the method is required to prevent non-targeted site introduction. Also, brain-specific mirna delivery is not yet successful 93, and effective neuron-specific delivery methods have to be developed to tackle brain and neuronal tumours. As discussed earlier, aav- and lentivirus-mediated delivery of let-7 in a mouse model of lung cancer 52,90 was found to be inefficient in pre-existing tumours because of the resistance to let-7 developed by the tumour over time 52. A strategy for let-7–mediated therapy for pre-existing tumours therefore also has to be developed.

### 2.6 Strategies to Overcome the Limitations

The optimal or normal level of let-7 may be restored in cancer cells either by administering exogenous let-7 *in situ* with a vector overexpressing let-7, or by repressing let-7 repressors. Recent mirna technologies are, in general, designed to use complementary or chemically modified single-stranded rna analogs (or both) to repress the specific mirnas responsible for a given disease or cancer. These analogs, including asos (antisense oligonucleotides), amos (anti-mirna asos called “antagomirs”), locked nucleic acids, and antisense-technology-based small interfering rnas, are widely and effectively used in regulation of mirna expression 92,94–99. But direct information is not available on the mirnas that regulate let-7 expression; this aspect limits the scope for such a strategy. Instead, technologies are required that can effectively upregulate let-7 expression. Hence, either vector-mediated overexpression of let-7 or transient transfection of double-stranded let-7 will be the choice.

Introduction of double-stranded let-7 duplex may produce mature let-7, equivalent to the endogenous version, during Dicer processing, potentially rescuing a downregulated let-7 level. This strategy has already been successfully used 83. Vectors containing pre–let-7–like synthetic short hairpin rnas, driven by highly inducible Pol iii promoters such as H1 and U6 100,101 may provide high expression of let-7 from predefined transcription start and termination sites 102. But instead of designing artificial hairpins, direct cloning of the entire natural pri–let-7 hairpin with flanking sequences into the expression vector may be a better approach— assuming that natural pre–let-7 will be a better substrate for generating mature let-7 during Dicer processing 103–107. A pri-mir–Pol ii transgene system has been successfully used to overexpress mir155 104, mir30 108, and mir122 109. This system was also found useful in expressing multiple mirnas from a single transcript 104 and can therefore be adopted for let-7 expression too.

High-density lipoprotein conjugated sirna has been reported to increase delivery efficacy in certain specific organs such as liver, gut, kidney, and steroid secreting organs 110. A similar approach may therefore have the possibility to be effective in let-7 delivery as well. But the synthesis and purification of therapeutic-grade let-7 is difficult. A nanoparticle-based delivery system may prove beneficial.

Other delivery methods that have been found promising in both *in vitro* and *in vivo* conditions include lentivirus-mediated pre–let-7 oligonucleotides 36, adenovirus-mediated delivery of hairpin sequences of mature let-7 90, cationic liposome–mediated delivery of pre–let-7 40, and electroporation of synthetic let-7 90. Although such methods are at the bench level, they might be translated into therapeutic approaches in the near future.

### 2.7 Current Industry Status of Let-7 Therapy

Because of its potential as a cancer therapeutic, let-7 has been filed for patent protection (Australia: 2007/333109 A1; United States: 20090163430). While diagnostic companies are developing let-7–based tests for various diseases, including several cancers, pharma giants are working toward development of effective delivery systems. But let-7 restoration methods are not yet satisfactory. Asuragen (www.asuragen.com), the rna-based therapeutic and diagnostics major with a core focus on mirna through its subsidiary Mirna Therapeutics (www.mirnatherapeutics.com), is developing mirna-based diagnostics and therapeutics for non-small-cell lung cancer, metastatic prostate cancer, and acute myeloid leukemia—all currently in preclinical trials. For lung cancer and acute myeloid leukemia, their main focus is let-7. Similarly, Regulus Therapeutics LLC (www.regulusrx.com) is using more than 60 mirnas, including let-7, to develop mirna therapeutics to treat several diseases (including cancers). Their main focus is on delivery systems and enhancement of treatment efficacy.

## 3. SUMMARY

Let-7 exerts its tumour suppressor and antiproliferative activities by repressing several oncogenes and by regulating key regulators of the cell cycle, cell differentiation, and apoptotic pathways. Downregulation of let-7 is a common phenomenon in several cancers, and restoration of normal let-7 expression has been found to prevent cancer growth. As a result, let-7 is a molecular marker in certain cancers and a potential therapeutic in cancer therapy. However, efficient delivery strategies have to be developed if this molecule is to be used as a therapeutic *in vivo.* Use of viral vectors, artificial virus-like particles, and nano materials may be a promising way to realize this goal, but optimization is needed. Also, a better understanding of let-7 biology and its regulatory networks is required to exploit the curative benefits of let-7 and to reduce off-target side effects.

## Figures and Tables

**TABLE I:** Deregulation of microrna let-7 family members in various cancers

Cancers	Microrna let-7 family members	References
Cancers that exhibit downregulation of specific let-7 family members		
Acute lymphoblastic leukemia	let-7b	Mi *et al.,* 2007 33
Bladder cancer	let-7b, let-7d, let-7e, let-7f	Nam *et al.,* 2008 34
Breast cancer	let-7, let-7a	Sempere *et al.,* 2007 35 Yu *et al.,* 2007 36
Bronchioloalveolar cancer	let-7	Inamura *et al.,* 2007 37
Burkitt lymphoma	let-7a	Sampson *et al.,* 2007 38
Colon cancer	let-7	Michael *et al.,* 2003 39 Akao *et al.,* 2006 40 Fang *et al.,* 2007 41
Gastric cancer	let-7	Zhang *et al.,* 2007 42 Motoyama *et al.,* 2008 43
Hepatocellular cancer	let-7	Johnson *et al.,* 2007 44
Kidney cancer	let-7a, let-7c, let-7d, let-7e, let-7f, let-7g	Nam *et al.,* 2008 34
Lung cancer	let-7	Johnson *et al.,* 2007 44 Takamizawa *et al.,* 2004 45 Johnson *et al.,* 2005 46
Malignant melanoma	let-7b	Schultz *et al.,* 2008 47
Ovarian cancer	let-7a-3	Lu *et al.,* 2007 48
Pancreatic cancer	let-7	Jérôme *et al.,* 2007 49
Prostate cancer	let-7c	Jiang *et al.,* 2005 50
Cancers that exhibit upregulation of specific let-7 family members		
Acute myeloid leukemia	let-7	Garzon *et al.,* 2008 51
Breast cancer	let-7b	Nam *et al.,* 2008 34
Colon cancer	let-7a, let-7g	Nam *et al.,* 2008 34
Lung cancer	let-7a	Nam *et al.,* 2008 34
Retinoblastoma	let-7a, let-7b, let-7c	Nam *et al.,* 2008 34
Uterine cancer	let-7i	Nam *et al.,* 2008 34

**Table II:** Microrna let-7 targets in various cancers

Cancer	Microrna let-7			Model used	References
	Expression	Targets	Effect on targets		
Breast cancer	let-7 ↓	*ANG; CCND1, 2; CDC25A; CDK4, 6;CYP19A1*; dna polymerases; *E2F5, 6; ESR1, 2; FGF11*; fgfr; *GRB2; HMGB2; IGF1, 1R; IL6; ITGB3; MAPK4, 6; MMP2; MMP8; MYC; ras; RB1; SKP2; TGFB1, BR1; TP53*	Transcription	*In silico*	Barh *et al.,* 2008 82
	let-7 ↓	*HMGA2,* H*-ras*	Transcription	Cell line, mouse model	Sempere *et al.,* 2007 35 Yu *et al.,* 2h007 36
Burkitt lymphoma	let-7a ↓	*MYC*	Transcription/translation	Cell line	Sampson *et al.,* 2007 38
Colon cancer	let-7 ↓	*ras*, *MYC*	Translation	Cell line	Akao *et al.,* 2006 40
Hepatocellular cancer	let-7 ↓	*AURKA; BRCA1, 2; BUB1; CCNA2, B1, E2, F, J; CDC2, 6, 20, 23, 25A, 34, 45L; NUF2; CBX2; CDCA2, 3, 4, 5, 7, 8; CDK8; CHEK1; CKS1B; DBF4; DICER1; E2F5, 6, 8;FANCD2; GMNN; CDT1; HMGA2; LIN28B; MAD2L1; NRAS; ORC1L; PLAGL1, 2; RRM1, 2; SKP2; SOX9; ARUKB* (formerly STK12)	Transcription	Cell line	Johnson *et al.,* 2007 44
Lung cancer	let-7 ↓	*MYC*, *ras*	Transcription/translation	Cell line	Johnson *et al.,* 2005 46 Kumar *et al.,* 2008 52
	let-7 ↓	*AURKA; CCNA2; CDC34; CDK8; DBF4; DICER1; E2F5; GMNN; HMGA2; LIN28B; NRAS; PLAGL1, 2; ARUKB* (formerly STK12)	Transcription	A549 lung cancer cells	Johnson *et al.,* 2007 44
	let-7 ↓	*HMGA2*	Transcription	Cell line	Kumar *et al.,* 2008 52 Lee and Dutta, 2007 83
Malignant melanoma	let-7b ↓	*CDK4*; cyclins A, D1, D3	Translation	Cell line	Schultz *et al.,* 2008 47
Uterine leiomyoma	let-7 ↓	*HMGA2*	Transcription	Tumour sample, cell line	Peng *et al.,* 2008 84

fgfr = fibroblast growth factor receptor; ↓ = downregulation.
